# Supplementary material for: Zinc is a key regulator of the sperm-specific K+ channel (Slo3) function
Source: eLife. 2026 Sep 30;14:RP105450. doi: 10.7554/eLife.105450 (PMC13626651; doi:10.7554/eLife.105450)
Supplement: Peer review history

# Editor report: eLife Assessment

**DOI:** 10.7554/eLife.105450.3.sa0

**Authors:** Anne E Carlson

**Affiliations:** University of Pittsburgh United States

**Keywords:** Solid, Valuable

This study presents a **valuable** finding on the role of intracellular zinc as a regulator of the sperm-specific potassium channel Slo3, demonstrating that zinc export during capacitation contributes to alkalinization-induced membrane hyperpolarization. The electrophysiological evidence supporting zinc-mediated inhibition of Slo3 is **solid**, though the mechanistic basis of this inhibition is not complete, as the proposed zinc-binding site involving E169 and E205 has not been directly tested through double-mutant analysis. This work will be of interest to reproductive biologists and ion channel biophysicists studying the molecular mechanisms of sperm capacitation.

# Referee report: Reviewer #1 (Public review)

**DOI:** 10.7554/eLife.105450.3.sa1

Summary:

In their manuscript, Andriani et al. show intracellular zinc is exported from sperm during capacitation and suppresses the alkalinization-induced hyperpolarization in sperm. Intracellular zinc inhibits Slo3 current, which is enhanced by the co-expression of gamma subunit Lrrc52. Computational studies reveal that the Zn binding site on mSlo3 is located near E169 and E205, which are involved in the sustained zinc inhibition of mSlo3 current. The authors propose that intracellular zinc play a key role of sperm capacitation by inhibiting the Slo3 channel.

Strengths:

Overall, the work appears well designed (e.g., oocyte patch-clamp experiments), and clearly presented. Three-dimensional structural modeling and flooding simulations are executed.

Weaknesses:

The simple mutagenesis analysis of E169 and E205 showed partial abolishment, but the molecular mechanism by which zinc inhibits Slo3 current is not yet fully shown. The authors should consider performing more extensive experiments, such as creating double mutants or combination mutants involving other residues. Additionally, could other mechanisms explain the role of zinc in regulating the Slo3 current?

While elucidating the mechanism of Slo3 is interesting, there is substantial literature indicating how zinc regulates channel functions at a molecular level. Given this, the manuscript should provide a deeper understanding by clearly elucidating the molecular mechanism of the regulation of Slo3 current by zinc.

The manuscript includes no experimental data on the mechanism of intracellular zinc export during sperm capacitation, despite being crucial for the regulation of sperm function.

# Referee report: Reviewer #2 (Public review)

**DOI:** 10.7554/eLife.105450.3.sa2

Summary:

In this paper, Andriani and colleagues are examining the potential role of Zn flux in sperm and its effect on Slo3 channels. This is an interesting question that is likely critical to how sperm function properly and Slo3 channels are a possible candidate for a downstream molecule that is impacted by Zn. In this paper the authors using Zn imaging, sperm motility assays, and electrophysiology to show that Zn flux has impacts on sperm function. They then go on to look at the impact Zn has on Slo3 current and propose a binding site based on MD simulations. Revisions of the paper added new critical controls and improved description of the methodology.

Strengths:

The question of how Zn flux impacts membrane potential and sperm motility is an important one. Moreover, Slo3 make present an interesting candidate or the target of Zn regulation. The combination of methods used here also has the potential to uncover mechanisms of Zn regulation of Slo3.

Weaknesses:

The responses sufficiently answered my original concerns.

# Author comment: Author response

**DOI:** 10.7554/eLife.105450.3.sa3

**Authors:** Rizki Tsari Andriani, Tanadet Pipatpolkai, Haruhiko Miyata, Masahito Ikawa, Yasushi Okamura, Takafumi Kawai

**Affiliations:** The University of Osaka Suita, Osaka Japan; Nanyang Technological University Singapore Singapore; The University of Osaka Osaka Japan; The University of Osaka Suita Japan; The University of Osaka Suita Japan; Ehime University Ehime Japan

The following is the authors’ response to the original reviews.

**Public Reviews:**

**Reviewer #1 (Public review):**

Summary:

In their manuscript, Andriani et al. show intracellular zinc is exported from sperm during capacitation and suppresses the alkalinization-induced hyperpolarization in sperm. Intracellular zinc inhibits Slo3 current, which is enhanced by the co-expression of gamma subunit Lrrc52. Computational studies reveal that the Zn binding site on mSlo3 is located near E169 and E205, which are involved in the sustained zinc inhibition of mSlo3 current. The authors propose that intracellular zinc plays a key role in sperm capacitation by inhibiting the Slo3 channel.

Strengths:

Overall, the work appears well-designed (e.g., oocyte patch-clamp experiments), and clearly presented. Three-dimensional structural modeling and flooding simulations are executed.

Weaknesses:

The simple mutagenesis analysis of E169 and E205 showed partial abolishment, but the molecular mechanism by which zinc inhibits Slo3 current is not yet fully shown. The authors should consider performing more extensive experiments, such as creating double mutants or combination mutants involving other residues. Additionally, could other mechanisms explain the role of zinc in regulating the Slo3 current?

We thank the reviewer’s thoughtful comments regarding the mutagenesis analysis and the possible mechanisms underlying zinc regulation of Slo3. Regarding the suggestion to perform double or combination mutants, we agree that such experiments would provide valuable mechanistic insight. However, due to limited resources, we were not able to perform these additional experiments within the scope of this study. Our current results show that mutations at E169 and E205 partially abolish zinc inhibition, which suggests that the inhibitory mechanism is not mediated through a single residue and is likely more complex.

Alternative mechanisms that may contribute to zinc modulation of Slo3 include indirect effects through modulation of nearby charged residues, structural rearrangements influenced by zinc binding, or the presence of multiple zinc binding sites within Slo3 channel other than the sites discovered through this study. At present, these mechanisms remain speculative and further studies will be required to clarify their contributions. This study provides the foundational basis for understanding how zinc inhibits the Slo3 channel and serves as an important starting point for defining the molecular mechanism in more detail.

We already acknowledged in the Discussion section that the precise molecular basis of zinc inhibition remains unknown and that future work involving more extensive mutational and structural analyses will be essential to fully resolve this issue.

We also added the discussion section as follows:

“It is worth noting that the incomplete loss of zinc sensitivity in these mutants suggests that additional mechanisms may participate in zinc modulation of Slo3. These may include modulation of nearby charged residues, structural rearrangements influenced by zinc binding, or the presence of multiple zinc binding sites. Comparisons with Slo2.2 (J. Zhang et al., 2023), KCNQ4 (Gao et al., 2017), and voltage-gated calcium channels (Sun et al., 2007) further support the possibility of diverse molecular determinants for zinc inhibition. Our VCF, mutagenesis, and simulation data together indicate that zinc influences voltage sensor movement in mSlo3, which may suggest a distinct inhibitory mechanism that warrants further investigation.”

While elucidating the mechanism of Slo3 is interesting, there is substantial literature indicating how zinc regulates channel functions at a molecular level. Given this, the manuscript should provide a deeper understanding by clearly elucidating the molecular mechanism of the regulation of Slo3 current by zinc.

Thank you for highlighting a very important point that requires deeper discussion and explanation regarding how zinc regulates Slo3 current at the molecular level. As reported, Slo3 is gated by membrane depolarization and, at the same time, this channel is also gated by intracellular pH, particularly alkalinization (Leonetti et al., 2012; Schreiber et al., 1998; X. Zhang et al., 2006). This makes the gating mechanism of this channel complex. The molecular mechanism underlying pH regulation of the Slo3 channel remains unknown (M. D. Lyon et al., 2023). We tested different pH conditions and membrane voltage to elucidate the effect of zinc on the Slo3 channel. Our data suggests that zinc inhibition in mSlo3 channels is dependent on pH (Fig. 2A-E), voltage (Fig. 2G-H; Fig.2—figure supplement 1A, B) and exhibits a long-lasting inhibitory effect (Fig. 2I, K).

However, as much as we are aware that these data alone cannot explain the molecular mechanisms of zinc’s effect on Slo3 current, our mutagenesis experiments also did not provide a straightforward answer. The single amino acid mutations examined in this study, which contain clustered negative residues, did not significantly alter zinc-mediated current reduction compared to the wild type. As the reviewer pointed out, mutating one single amino acid may not be sufficient to fully identify other contributing residues within the predicted mSlo3 zinc-binding site. Therefore, more extensive mutagenesis studies will be required to fully elucidate the molecular mechanism of zinc inhibition in mSlo3, which could not be fully understood in this study.

On the other hand, when we analyzed the percentage of current recovery of all the mutants, E169A and E205A showed significant current recovery upon the wash-out by pH 8.0 alone. Consistent with MD simulations, our electrophysiological recordings demonstrated that the long-lasting inhibitory effect of zinc was partly abolished by these mutations. Thus, our findings highlight the contribution of E169A, located at the lower end of S3 domain and E205A, located at the lower region of S4 domain, to zinc-mediated inhibition of mSlo3 current.

Additionally, since the molecular mechanism of pH regulation on Slo3 channel remains unknown, the molecular basis of its dual gating has yet to be elucidated, making it difficult to draw a single definitive conclusion from our current research data on how zinc inhibits mSlo3 current. Nevertheless, this study provides the foundation for understanding possible mechanisms of zinc inhibition. Our VCF data suggest that zinc influences the movement of VSD of mSlo3, and together with our mutagenesis and MD simulations results, these findings represent an important first step toward elucidating the molecular mechanism of zinc inhibition of the mSlo3 current.

Intracellular zinc exerts inhibitory effect on mSlo3, similar to what has been reported for Slo2.2 channels (J. Zhang et al., 2023), high- and low-voltage activated calcium channel families (Sun et al., 2007) and KCNQ4 channels (Gao et al., 2017). These studies identified different regions, amino acids, and possible mechanisms of zinc inhibition among these ion channels. For instance, in Slo2.2 channels, which belong to the same Slo family as Slo3, the zinc-binding site was identified in the RCK2 domain, where cysteine and histidine residues form a canonical zinc binding motif (J. Zhang et al., 2023). In KCNQ4 channels, zinc inhibits the channel activity in a non-canonical manner that depends on its physiological activator, the membrane lipid PI(4,5)P_2_ (Gao et al., 2017). Although zinc exerts the inhibitory effects on those various voltage-gated potassium and calcium channels, the mechanisms differ. Our data suggests another distinct mechanism of zinc inhibition in the mSlo3 channel with the identified sites located in the VSD, where zinc influences the voltage-sensor motion, and consequently affects the complex gating of Slo3.

We revised the discussion section as follows, which is also related to the previous comment:

“It is worth noting that the incomplete loss of zinc sensitivity in these mutants suggests that additional mechanisms may participate in zinc modulation of Slo3. These may include modulation of nearby charged residues, structural rearrangements influenced by zinc binding, or the presence of multiple zinc binding sites. Comparisons with Slo2.2 (J. Zhang et al., 2023), KCNQ4 (Gao et al., 2017), and voltage-gated calcium channels (Sun et al., 2007) further support the possibility of diverse molecular determinants for zinc inhibition. Our VCF, mutagenesis, and simulation data together indicate that zinc influences voltage sensor movement in mSlo3, which may suggest a distinct inhibitory mechanism that warrants further investigation.”

The manuscript includes no experimental data on the mechanism of intracellular zinc export during sperm capacitation, despite being crucial for the regulation of sperm function.

We thank the reviewers for the valuable comment in this regard. We agree that mechanism of intracellular zinc export during capacitation is crucial for the regulation of sperm function, and it would be an important finding if we could provide the experimental data on this. However, there are significant technical difficulties in performing such experiments. Two protein families facilitate the transport of zinc across cellular and intracellular membranes in opposite directions: ZnT and ZIP. ZIP12 has been reported to be highly expressed in mouse testis (Zhu et al., 2022), as well as ZnT-1 (Elgazar et al., 2005). To date, there are no known inhibitors for zinc transporters, and there is also no suitable antibodies available for these transporters, which makes it difficult to design experiments to examine the intracellular zinc transport during sperm capacitation. Apart from the two reported zinc transporters, the functional significance of other ZnTs and ZIPs, particularly those related to capacitation, remains largely unclear, leaving the mechanisms of zinc transport in sperm during capacitation poorly understood. Moreover. homozygous Znt-1 knockout mice exhibit a lethal phenotype (Andrews et al., 2004).

**Reviewer #2 (Public review):**

Summary:

In this paper, Andriani and colleagues are examining the potential role of Zn flux in sperm and its effect on Slo3 channels. This is an interesting question that is likely critical to how sperm function properly and Slo3 channels are a possible candidate for a downstream molecule that is impacted by Zn. In this paper, the authors use Zn imaging, sperm motility assays, and electrophysiology to show that Zn flux impacts sperm function. They then go on to look at the impact Zn has on Slo3 current and propose a binding site based on MD simulations. While the ideas are interesting, the experiments are not well described in many places making understanding the results very difficult. In addition, critical controls are missing throughout the paper.

Strengths:

The question of how Zn flux impacts membrane potential and sperm motility is an important one. Moreover, Slo3 presents an interesting candidate or the target of Zn regulation. The combination of methods used here also has the potential to uncover mechanisms of Zn regulation of Slo3.

Weaknesses:

Much of the paper lacks experimental description which makes interpretation quite difficult, or a detailed discussion is missing. Examples include:

(1) Figure 1, particularly the Zn imaging, is not sufficiently described. How is the fluorescence intensity measured? A representative ROI? The whole tail and head? Are the sperm immobile? If not, there is evidence that motion artifacts can significantly distort these sorts of measures from Calcium measurements in Cilia. Were there controls done? Is the small amount of Zn seen in the tail above the background?

We sincerely thank the reviewer for pointing out important details that we should provide in this study in order to make it well understood. We would like to answer and respond to the points raised by reviewer as follows:

Fluorescence intensity is measured by the signal taken from the whole head and the proximal part of tail in sperm. We have included this in the materials and methods.

Materials and Methods

“Fluorescence intensity is measured by the signal taken from the whole head and the proximal part of tail in sperm.”

Yes sperm is immobile during zinc imaging.

We added the control data of zinc imaging without capacitation medium and incorporated the data into the graph in Figure 1B. For the control in non-capacitation medium, we use HS medium as newly explained in the methods, results, related figure (Figure 1B), and figure legends.

Yes the small amount of Zn seen in the tail above the background. As shown in Fig. 1A we confirmed that the signal intensity at the proximal region of the tail was higher than the background. Therefore, the data for this region were calculated after background subtraction.

(2) The second half of Figure 1 is also not well described. What is the extracellular solution in the recordings? When you apply the Zn ionophore, do you expect influx or efflux? I assume efflux is based on the conclusions but this should be discussed explicitly.

The extracellular solution in the recordings for Figure 1 is HS solution (HEPES-buffered saline solution), a standard non-capacitation medium. We will include this information in the materials methods.

Materials and methods

“HS-based solution was used as the extracellular solution.”

We assume that intracellular zinc levels increase upon application of zinc ionophore. Previous work has reported that sperm contain approximately 35.7 ng/10^6^ cells in the head and flagellum (Henkel et al., 1999). When zinc pyrithione is applied, it facilitates the influx of Zn^2+^ from the surrounding medium into the cell, thereby increasing intracellular zinc concentration. Zinc pyrithione functions both as a zinc source and as a transport facilitator, allowing Zn^2^ to cross the otherwise impermeable lipid membrane without compromising membrane integrity.

(3) Figure 2H labels the Y axis, "normalized current". Normalized to what? Why do neither of the curves end at 1? A better description of what this figure represents is needed.

Normalization for figure 2H was performed by dividing the absolute current of mSlo3 at pH 8.0 of each voltage by the absolute current at the pre-determined highest voltage that still produced a stable mSlo3 current (i.e., good patch, good clamp). In this analysis, +140 mV was chosen as the highest voltage for normalization, since in few cells the patch was lost at +160mV and +180mV. Similar to the control condition, the absolute current of mSlo3 in the presence of 100 µM zinc was normalized to the absolute current of the control at +140 mV. This information has been included in the figure legends and the Materials Methods section of the revised manuscript.

Materials Methods section:

Figure legends for figure 2H has been updated.

(4) The alpha fold simulations are not well described. How many Zn binding sites were found? Are all of the histidine mutations in Figure 4 Supplement 1 the ones that were found?

We thank the reviewer for the question. In our AlphaFold3 input, we only input the transmembrane region of the protein. From there, we found four sites located as follows:

Given that we are only interested in the intracellular side of the membrane, we are only interested in the site with the highest pLDDT value (confidence values). On the IC side, there are only two sites, where the other sites are located near the pore domain. The site is near E310 and K319.

**Author response image 1.** AlphaFold3 prediction of the Zn binding site on IC side of Slo3.

The histidines in Fig. 4—figure supplement 1 are all histidines that are not in the transmembrane region. These residues were not included in the initial inputs for AlphaFold3. However, we conducted MD simulations including these residues and we were able to show that a few of these residues are in contact with Zn. We have now plotted the minimum distance between each of these residues and Zn in the flooding simulations.

**Author response image 2.** MD simulations of histidines residues located in IC of Slo3.

Minimum distances between histidines in Fig. 4—figure supplement 1 and Zn^2+^ from the flooding simulations. Different colors indicate different repeats.

(5) There is no discussion of physiological intracellular Zn concentration. How much Zn is inside the sperm? How much if likely Free vs buffered? Is 100uM a reasonable physiological concentration?

We estimated the intracellular zinc concentration in sperm based on human sperm data, which report a zinc concentration of approximately 35.7 ng/10^6^ cells in the head and flagellum (Henkel et al., 1999). Considering the volume of a typical human sperm is about 15 µm^3^ (Laufer et al., 1977), this translates to an estimated intracellular zinc concentration of approximately 400 mM, although the concentration of free zinc must be much lower than this level. Although exact intracellular zinc concentrations in mouse sperm are not well-documented, this estimate supports the observation of elevated zinc in non-capacitated sperm.

There are a number of areas where the interpretation is not well supported by the data including:

(6) You say in the Figure 4 supplement, that "we did not observe any significant decrease in the percentage of current inhibition." But that is a pretty misleading statement. There are large changes (increases) in the amount of zinc inhibition. These might be allosteric changes but I don't think you can safely eliminate these as relevant Zn binding sites. Also, some of these mutations appear to allow at least some unbinding of Zn.

In our MD simulations, H720 is not at the zinc binding site and therefore, mutation to arginine would indeed eliminate its binding. We are showing this in the minimum distance analysis between Zn and H720 and show that they are further than 4 Å from each others (n=3), as shown in author response image 2.

Chimera of Slo3/Slo1 RCK2 also showed large increases in the amount of zinc inhibition, and this might serve as a potential binding site. We agree that the statement: “we did not observe any significant decrease in the percentage of current inhibition.” is misleading, therefore we revised our interpretation and statement into:

We revised the result section as follows:

“However, the percentage of current inhibition varied across the mutated constructs, showing either increases or no appreciable change (Fig. 4—figure supplement 1B, C).”

(7) Following up on the above point, it seems unfair to conclude that the D162S, E169A, and E205 mutants are part of the inhibitory binding site for Zn when the mutation has no effect on inhibition and only an effect on the washout. The mutations on the intracellular side also had an impact on the washout so it seems equally likely that they are the critical residues based on your data.

We thank the reviewer for this important point. We agree that the absence of a strong reduction in the initial zinc inhibition makes it challenging to assign any single residue as a definitive zinc binding site. However, our interpretation is based not only on the electrophysiological data but also on the MD simulations, which consistently identified E169 and E205 as residues that frequently interact with zinc and stabilize zinc occupancy within the VSD region. Although the mutations did not markedly reduce the peak level of zinc inhibition, both E169A and E205A significantly altered the long-lasting inhibitory component during washout, which is consistent with the MD-predicted interactions. In contrast, the intracellular mutations affected washout but were not supported by MD simulations as potential zinc interaction sites. Taken together, these combined datasets support the idea that E169 and E205 contribute to zinc modulation of Slo3 in the VSD, even though additional residues or mechanisms are likely involved.

(8) Nowhere in the paper do you make the specific link between Zn flux and membrane hyperpolarization via Slo3. You show that Zn flux changes the ability of the sperm to hyperpolarize and you show that Slo3 is inhibited by Zn but the connection between the two is not demonstrated. There appears to be a specific Slo3 blocker. If you use this in sperm, do you no longer see the Zn effect?

Thank you for pointing out the need for clarifying this point. It is already known that sperm capacitation is well associated with the increase of intracellular pH (Vredenburgh‐Wilberg & Parrish, 1995; Y. Zeng et al., 1996), the hyperpolarization of the membrane (Arnoult et al., 1999; Y. Zeng et al., 1995) and the elevation of intracellular Ca^2+^ concentration level (Breitbart, 2002; Publicover et al., 2007) through diverse ion channel activities. To explore whether these pathways are influenced by intracellular zinc, we used patch-clamp techniques to measure the membrane potential (Vm) as shown in Fig. 1D-K. It has been reported that under the whole-cell current clamp of mouse epididymal spermatozoa, resting membrane potential is hyperpolarized after intracellular alkalinization (Navarro et al., 2007). We mentioned this in line 100-108 in the manuscript.

Next, our findings from the experiments using mouse spermatozoa suggest that intracellular zinc inhibits a key process in sperm capacitation, specifically the alkalinization-induced hyperpolarization. Previous studies have identified the pH-and voltage-dependent potassium channel Slo3 is responsible for the principal K^+^ current (I_KSper_) in mouse spermatozoa (Navarro et al., 2007; Santi et al., 2010; Schreiber et al., 1998; X. H. Zeng et al., 2011). During capacitation, the rise in pHi leads to the activation of Slo3 channels, resulting in membrane hyperpolarization (Santi et al., 2010). Given this context, we next investigated whether intracellular zinc acts directly on the Slo3 channel and found that zinc inhibits mSlo3 current. We explained this rationale of the experiment in line 143-150.

We add following sentence to add more clarity to the text:

“During capacitation, the rise in pHi leads to the activation of Slo3 channels, resulting in membrane hyperpolarization (Santi et al., 2010).”

Therefore, the text was modified into:

“Our findings suggest that intracellular zinc inhibits a key process in sperm capacitation, specifically the alkalinization-induced hyperpolarization. Previous studies have identified the pH-and voltage-dependent potassium channel Slo3 is responsible for the principal K^+^ current (I_KSper_) in mouse spermatozoa (Navarro et al., 2007; Santi et al., 2010; Schreiber et al., 1998; X. H. Zeng et al., 2011). During capacitation, the rise in pHi leads to the activation of Slo3 channels, resulting in membrane hyperpolarization (Santi et al., 2010). Given this context, we next investigated whether intracellular zinc acts directly on the Slo3 channel.”

Regarding the specific inhibitor, as has been pointed out by the reviewer that a new Slo3 inhibitor, VU0546110, exhibited more than 40-fold selective for human Slo3 over Slo1 (M. Lyon et al., 2023). However, the effect of VU0546110 in mSlo3 has not been tested yet. Both mouse and human Slo3 exhibit similar responses to certain inhibitors, but mouse and human Slo3 is also differ in their responses to several other inhibitors (M. D. Lyon et al., 2023), making it uncertain if this VU0546110 will work on mSlo3.

(9) In the second half of Figure 1, the authors suggest that there is "no hyperpolization in 100uM Zn. That is not really true. It is reduced but not absent.

We modified the wording of “no hyperpolarization in 100 µM Zn” to “alkalinization-induced hyperpolarization was reduced in the 100 µM ZnCl_2_ group.”

“In contrast, alkalinization-induced hyperpolarization was reduced in the 100 µM ZnCl_2_ group”

(10) The claim that Lrcc52 with Slo3 shows a higher current inhibition at pH 7.5 than pH 8 is not well supported because there are only 3 replicates in the 7.5 case. In addition, the claim is made in the test that 100uM ZnCl2 "already inhibited mSlo3+Lrcc52 at pH7.5", contrasted with mSlo3 alone, is not tested statistically.

Thank you for the valuable comment. Although Fig. 3F shows a statistical difference, we agree that having only three replicates at pH 7.5 may somewhat weaken the conclusion. Following this suggestion, we have revised the sentence as follows:

“Alkalinization appeared to increase the percentage of current inhibition by 100 µM ZnCl_2_.”

We provided statistical analysis to compare pH 7.5 between mSlo3 alone and mSlo3+Lrrc52 in the Figure 3—figure supplement 1D:

The statistical analysis showed that 100 µM zinc significantly inhibited the mSlo3 + Lrrc52 current at pH 7.5 compared to the mSlo3 current alone. We have incorporated the necessary changes into the revised manuscript and updated the figure legends accordingly.

In a number of places, better controls are needed.

(11) How specific is this effect for Zn? Mg2+, for instance, is also a divalent cation that is in the hundreds of uM range inside the cell. Does it exert the same effect? Each ion certainly has unique preferred coordination geometries, does your predicted binding with MD show what you might expect for tetrahedral coordination with Zn? Did you test other divalent cations functionally or in silicon?

To answer this question, we have tested this by building another AlphaFold3 model, with Mg^2+^ instead of Zn^2+^. We did not opt for the all-atoms MD simulations due to the cost of the simulation. Here, the model shows that Mg are all clustered at the pore domain and does not reside anywhere near the Zn^2+^ site from both MD simulations and the AF3 model.

**Author response image 3.** AlphaFold3 model of Slo3 channel with Mg^2+^.

The Slo3 AlphaFold model from residue M1 to L330. The colour gradient reflects the pLDDT score range from 1.73 to 95.69. Purple sticks highlighted E169, N171 and E205. In this study, we did not examine other divalent cations in our electrophysiological recordings. Exploring their effects will be an important direction for future research.

(12) For the VCF experiments, a significantly higher concentration of Zn was used (10mM). What is the reason for this? There is no discussion of how much a "puff" is. Assuming you are using the RNA injector it is probably on the order of 50nL or less. Assuming the volume of an oocyte is 1uL that would argue that the final concentration is 500uM or higher. But this is also complicated by potential local effects of high Zn at the injection site, artifacts of injecting that much metal, and the fact that a great deal of the Zn will likely be bound to other things inside the cell. Better controls are needed for this experiment.

As pointed out by the reviewer, the volume of the oocytes is estimated to be approximately 1 µL. We performed manual injections using glass needle typically used for RNA injection. However, because the injections were done manually during real-time VCF recording (as illustrated in the experimental scheme), the exact volume of the solution injected into each oocyte could not be precisely controlled. We estimated that each drop to be approximately 50 nL, resulting in a final concentration around 500 µM, as described by the reviewer.

The rationale for using relatively high concentration was to ensure that the zinc concentration inside the oocyte reached an effective level, since manual injection may sometimes deliver less than 50 nL of solution. In some cases, injections failed entirely due to the technical difficulty of the method. Because VCF recordings are already technically difficult, we aimed to ensure that zinc injection was successful in oocytes that exhibited robust fluorescence signal by injecting an excess amount of zinc that would not disrupt normal oocyte conditions. For example, 10 mM zinc was prepared in an acidic solution (pH 2.5). We verified that this acidic condition did not affect mSlo3 current by performing control injections with the acidic solution alone, since the mSlo3 current is not activated under acidic pH conditions

**Author response image 4.** VCF control experimentes: vehicle injection.

**Reviewer #3 (Public review):**

Summary:

The study titled "Zinc is a Key Regulator of the Sperm-Specific K+ Channel (Slo3) Function" aims to investigate the role of intracellular zinc in sperm capacitation and its regulation of the sperm-specific Slo3 potassium channel. Capacitation is a crucial physiological process that enables sperm to fertilize an egg, and membrane hyperpolarization through Slo3 activation is a well-established event in this process. The authors propose that intracellular zinc dynamically decreases during capacitation and inhibits Slo3-mediated K⁺ currents, thereby playing a regulatory role in sperm function.

Strengths:

(1) Novel Contribution to Sperm Physiology.

The study provides new insights into how zinc dynamics contribute to sperm capacitation, specifically through its direct inhibition of Slo3 activity.

Previous research has focused primarily on extracellular zinc's effect on sperm function; this work expands the discussion to intracellular zinc regulation, an area with limited prior investigation.

(2) Strong Electrophysiological Evidence.

The study employs inside-out patch-clamp recordings in Xenopus oocytes to demonstrate zinc's direct inhibition of Slo3 currents. The observed slow dissociation of zinc from Slo3 suggests a long-lasting regulatory effect, adding to the understanding of ion channel modulation in sperm cells.

(3) Molecular Mechanistic Insights

Using Molecular Dynamics (MD) simulations and mutagenesis, the authors identify potential zinc-binding sites within Slo3's voltage-sensing domain (VSD), particularly E169 and E205. These computational predictions are supported by electrophysiological recordings, strengthening the argument that zinc directly binds and inhibits Slo3.

(4) Physiological Relevance and Functional Implications

The study suggests that zinc inhibition of Slo3 could contribute to sperm motility regulation during capacitation.

The authors provide sperm motility assays as supporting evidence, showing that zinc chelation affects motility only after capacitation has begun, suggesting a dynamic role of intracellular zinc in the capacitation process.

Weaknesses:

While the study presents compelling electrophysiological data and molecular insights, there are several critical gaps that must be addressed before fully supporting the physiological relevance of the findings.

(1) The authors should measure the effects in sperm cells using the patch-clamp technique to directly record Slo3 currents. By normalizing Slo3 currents to cell capacitance at different intracellular zinc concentrations, the authors can quantitatively assess the extent of Slo3 inhibition by zinc and strengthen the physiological relevance of their findings.

We thank the reviewer for the valuable comments to strengthen the physiological relevance of our findings. We provided additional data of Slo3 currents measured using perforated patch-clamp recording in sperm cells in experiments with zinc pyrithione (ZnPy) before and after the addition of 10 mM NH_4_Cl. Control experiments were conducted in the absence of ZnPy, in which Slo3 current were recorded before and after the application of 10 mM NH_4_Cl. These data have been integrated into Figure 1L-N and Figure 1—figure supplement 1A, B.

It is worth noting that Slo3 current in this recording might contain other endogenous current, as no specific blocker was used. Nonetheless, the data showed that the Slo3 current in sperm tends to be inhibited by zinc, as shown by the plot of absolute Slo3 current after the addition of 10 mM NH_4_Cl in the absence of ZnPy (control) and in the presence of 100 µM ZnPy. There was a decrease in the fold change calculated from the absolute current before and after the addition of 10 mM NH_4_Cl of ZnPy treated group compared to the control group.

We also provided data with the cell capacitance as suggested; however, cell capacitance obtained from the sperm recordings showed the capacitance throughout the head and midpiece of spermatozoa. On the other hand, Slo3 channels are not expressed in the entire spermatozoa, therefore the cell capacitance acquired from these recordings does not accurately reflect the area where the Slo3 channels are localized. Although we included normalization of Slo3 currents to cell capacitance before and after ZnPy application, this normalization should be interpreted with caution for the reasons mentioned above. The corresponding figure has been included in the supplementary data Figure 1—figure supplement 1A, B.

We added sentences to the result section as follows:

“We also measured Slo3 current using perforated patch-clamp recordings in spermatozoa treated with ZnPy, before and after the addition of NH_4_ Cl. Control experiments were conducted in the absence of ZnPy, in which Slo3 current were recorded before and after the application of 10 mM NH_4_Cl (Fig. 1L-N; Fig. 1—figure supplement 2A, B). Slo3 current in sperm tended to be inhibited by zinc, as shown by the plot of absolute Slo3 current after the addition of 10 mM NH_4_Cl in the absence of ZnPy (control) and in the presence of 100 µM ZnPy (Fig. 1L, M). There was a decrease in the fold change calculated from the absolute current before and after the addition of 10 mM NH_4_Cl of ZnPy treated group compared to the control group (Fig. 1N). Taken together, these results confirmed that intracellular zinc indeed inhibits alkalinization-induced hyperpolarization in mouse sperm.”

(2) Lack of Controls in Non-Capacitated Sperm

The claim that zinc is exported from sperm during capacitation needs stronger experimental validation.

The authors did not include a control group of non-capacitated sperm in key fluorescence imaging experiments, making it difficult to confirm that the observed zinc decrease is capacitation-specific rather than a general zinc redistribution process.

To strengthen this conclusion, experiments should be performed in non-capacitating conditions to determine whether intracellular zinc levels remain unchanged.

We added the control group of non-capacitated sperm in key fluorescence imaging experiments, as integrated in Figure 1B.

The following changes in the Results and Figure Legend sections are revised and added:

“We observed that there was a gradual and significant decrease in fluorescence intensity in both regions (Fig. 1B), particularly prominent in the flagellum (Fig. 1C). This decline suggests the active release of intracellular zinc from sperm flagellum occurs during capacitation. In contrast, the fluorescence intensity of the control group of non-capacitated sperm remained unchanged (Fig. 1B).”

Figure Legend 1B was modified accordingly.

(3) Unclear Role of Zinc in Physiological Capacitation

The study clearly demonstrates zinc inhibition of Slo3 but does not sufficiently establish how this affects capacitation at a functional level.

Additional motility and capacitation markers should be analyzed to confirm that zinc influences sperm behavior beyond Slo3 inhibition.

We thank the reviewer for this valuable comment. We fully agree that zinc can influence sperm physiology through multiple mechanisms and that its overall effects on capacitation are complex. However, the main goal of our study is to investigate the mechanism and to determine whether intracellular Zn^2+^ directly inhibits Slo3. Our results from both the heterologous expression system and the sperm membrane potential recordings consistently support this conclusion.

For these reasons, we believe that adding such assays would not clarify the role of Slo3 in capacitation but rather risk confounding interpretation. Instead, we have expanded the Discussion to explicitly acknowledge these limitations and to emphasize that future studies combining genetic or pharmacological modulation of Slo3 with comprehensive capacitation analyses will be required to fully define its physiological impact.

We added sentences to the discussion section in the revised manuscript as follows:

“Although these results support a mechanistic link between zinc and Slo3 activity, future studies that combine genetic or pharmacological modulation of Slo3 with comprehensive capacitation analyses will be required to define its physiological impact in more detail. Within this context, this study highlights the potential importance of intracellular zinc in the regulation of sperm capacitation.”

(4) Insufficient Data on Zinc-Slo3 Specificity

The authors should consider using quinidine, a known washable Slo3 inhibitor, to confirm that zinc acts specifically on Slo3 channels rather than other endogenous ion channels.

The study would benefit from including washout controls in the inside-out patch-clamp recordings, as seen in Figure 3-Supplement 1, to confirm that zinc inhibition is reversible or long-lasting.

We thank the reviewer for raising the point regarding the need to confirm that the current observed in our recordings indeed represents Slo3 current by using a specific blocker such as quinidine, as there is a possibility that endogenous currents might also be present and that zinc could act on those endogenous currents. Performing experiments with quinidine would indeed be crucial to demonstrate the specificity of Slo3 current in our patch-clamp recordings.

However, in our current experimental protocol, we apply ramp pulses multiple times and require a long series of recordings within a single session in one patch as described in the materials and methods as well as Figure 2I, Figure 4—figure supplement 1C, Figure 5B (pH 8.0 → 100 µM zinc → pH 8.0, to observe the washout effect). Incorporating quinidine into this sequence would make the protocol even longer (pH 8.0 → quinidine → washout → pH 8.0 → 100 µM zinc), which increases the likelihood of patch loss before completing the full set.

Furthermore, we have ensured that the recorded current corresponds to Slo3 by using appropriate experimental conditions, specifically the suitable voltage range for activation, a high intracellular pH (pH 8.0), and high-potassium solutions in our recordings.

(5) Missing Discussion of Zinc's Role in CatSper Regulation

The study focuses solely on Slo3 but does not mention CatSper, the principal Ca^2+^ channel essential for sperm capacitation.

Zinc has been reported to inhibit CatSper activity, which could significantly impact sperm function.

The discussion should address whether zinc's effect on Slo3 represents a broader regulatory mechanism influencing multiple ion channels during capacitation.

Thank you for the comment. To the best of our knowledge, there have been no reports showing that CatSper activity is directly regulated by zinc ions.

Furthermore, in our patch-clamp recordings with NH_4_Cl and ZnPy, we observed that the normal CatSper current increased even in the presence of ZnPy, which makes it challenging to conclude whether zinc directly affects CatSper channel activity.

We added sentences to the discussion section in the revised manuscript as follows:

“In addition to that, to date, there are only few reports on the effect of zinc on other sperm ion channels, and none have been reported in mouse sperm. One important study was reported by (Jeschke et al., 2021), in which seminal zinc was found to inhibit prostaglandin-induced activation of CatSper, a sperm-specific Ca^2+^ channel, in human sperm. The complex opposing action of seminal zinc and prostaglandins on CatSper may help preventing premature activation of CatSper in the ejaculate and act as a dilution sensor, although this study does not provide direct evidence for zinc acting directly on CatSper (Jeschke et al., 2021).”

Final Assessment

This work presents important findings on zinc regulation of Slo3 channels, supported by strong electrophysiological and molecular analyses. However, the physiological relevance of these findings remains unclear due to missing controls, and needs additional functional assays. Addressing these issues would significantly enhance the manuscript's scientific rigor and impact.

**Recommendations for the authors:**

**Reviewer #2 (Recommendations for the authors):**

Most of the specific comments and suggestions are in the public review. Minor additional comments primarily focused on presentation and textual errors are here.

(1) There is something strange happening in Figure 6D in the -100ish range. I think it's likely related to the reversal potential of K+.

Thank you for pointing it out. Yes in figure 6D there was strange plot in the range of -100 mV. As the reviewer has pointed out we also think that it is related to the reversal potential of potassium ions.

(2) There are a number of errors in the text that make following it difficult. For instance, multiple times the authors say "In consistent" (line 120 as an example) when I think they mean consistent with.

We changed the “in consistent” with “consistent with” throughout the revised manuscript.

**Reviewer #3 (Recommendations for the authors):**

The authors provide well-described experiments, particularly those examining the effects of intracellular zinc on Slo3 channels using inside-out patch-clamp recordings. However, some experimental designs intended to assess the physiological relevance of these findings during capacitation require additional controls and data before the authors' claims can be fully supported.

Comments

Major Concerns & Suggested Improvements

Line 65: "In the present study, we find that intracellular zinc is exported during capacitation, indicating that zinc dynamics in spermatozoa play an important role in fertilization."

This claim requires additional experimental data to be fully supported.

Thank you for pointing it out. We have provided data for control experiments of zinc imaging in non-capacitated conditions in Figure 1B.

Line 79: "Intracellular zinc is exported from sperm during capacitation."

The authors should include controls in non-capacitated conditions to determine whether zinc export is specific to capacitation or a general process in sperm cells.

Again, we have provided data for control experiments of zinc imaging in non-capacitated conditions in Figure 1B.

Figures - General Comment:

In all figures, please replace SEM (Standard Error of the Mean) with Standard Deviation (SD) for consistency and a more accurate representation of variability.

SEM (Standard Error of the Mean) has been replaced with SD (Standard Deviation) in all figures (main figures and supplements) as well as in numerical description accordingly.

Figure 1

Panel B:

Include a non-capacitating media control to confirm that the observed decrease in zinc-sensitive dye fluorescence is not due to artifact/photobleaching.

We have provided data for control experiments of zinc imaging in non-capacitated conditions in Figure 1B.

Perform an experiment with capacitating media supplemented with a higher concentration of zinc. If intracellular zinc export is a real effect, added extracellular zinc should prevent or reduce this phenomenon.

We appreciate the reviewer’s suggestion; however, we believe that supplementing the medium with high concentrations of zinc is unsuitable for validating the export phenomenon due to confounding physiological factors. Our preliminary tests demonstrated that increasing extracellular zinc triggers a drastic increase in intracellular zinc as well (Author response image 5). Furthermore, the high concentration of BSA in the capacitation medium acts as a potent zinc buffer, precluding precise control over free Zn^2+^ levels. Therefore, the inherent difficulty in maintaining defined extracellular and intracellular Zn^2+^ gradients makes the interpretation of such data highly problematic. Future studies will focus on identifying the specific zinc transporters involved and characterizing their molecular mechanisms.

**Author response image 5.** Zinc addition.

Clarify whether the "n" value represents different cells or multiple recordings from the same cell.

n value represents different cells.

Supplemental Figure 1:

Incorporate Δ (delta) comparison between 10 min and 2 hours under control conditions and in the presence of TPEN.

Here we provide data:

**Author response image 6.** Δ comparition between control and TPEN.

Provide statistical analysis for these comparisons to make the effects of capacitation clearer.

We did the calculation and statistical analysis, however there was no statistical difference, as shown in the author response figure 6 due to high variability of individual data.

Figure 2

Panel C:

Incorporate inhibition at pH 7.4 and 6.0 for direct comparison.

Recording inhibition effect of zinc at pH 6.0 is not possible because there would be no current to begin with, as mSlo3 is gated by both voltage and alkaline pH.

Panel D:

Include a washout control, similar to what is shown in Panel A.

We included a washout control trace to Figure 2D.

Panel E:

Provide a longer reference trace in the absence of zinc to clearly visualize the control condition. The current reference segment is too short to properly assess baseline activity.

Although we do not have a longer reference trace in the absence of zinc for Figure 2E, we instead show the trace recorded under the application of 0.1 µM zinc in Figure 2—figure supplement 1A to illustrate the current behavior.

Panels G-H:

Include inside-out patch-clamp traces and quantification of zinc washout effects.

Inside out patch traces are shown in Figure 2G as we applied step-pulses protocol. The zinc washout effect could not be quantified because the patch was usually lost after the second step-pulse application.

Panels I-K:

Provide additional traces. In Panel I, the inhibition by zinc is clear, but in Panel J, the reduction appears less distinct and could be due to rundown or an artifact. Additional controls should clarify this.

Figure 2K presents the most representative trace among five recorded cells. The apparent reduction is less distinct, likely due to an artifact caused by a bubble in the rapid perfusion system during solution exchange. However, at the end of zinc application (*t* = 50 s), the current amplitude was clearly reduced compared with that at *t* = 0–10 s.

Figure 3

Panel D:

Include additional data showing the transition to pH 6 and washout with pH 7.5, similar to the experimental design in Panels A and B.

We included additional data showing raw trace of the application of pH 6.0 in Figure 3D, also included the transition to pH 6 and washout with pH 7.5 in Figure 3E.

Figure 3-Supplement 1:

Include zinc washout experiments. This approach is one of the best ways to evaluate the reversibility of zinc inhibition on the channel.

As mentioned above, in this recording we recorded step pulses up to +180 mV. The zinc washout effect could not be quantified because the patch was usually lost after the second step-pulse application.

Figure 6

Zinc Inhibition Specificity:

The authors should use quinidine, a known washable Slo3 inhibitor, to assess Slo3 activity before and after zinc injection.

This experiment would confirm that zinc specifically inhibits Slo3, rather than affecting other endogenous channels.

We sincerely thank the reviewer for this valuable suggestion. However, given the technical difficulty of these experiments, which involve lengthy VCF recordings and manual zinc injections that significantly compromise oocyte health, it is not feasible to apply quinidine at this stage.

Moreover, we observed voltage-dependent fluorescence changes around the VSD, and this change was influenced by the application of zinc, confirming that zinc specifically inhibits Slo3 rather than affecting other endogenous channels.

Discussion - Key Revisions Needed

Line 308: "Our results demonstrated that intracellular zinc is exported from spermatozoa during capacitation."

This claim needs to be supported by experiments using non-capacitated conditions.

Additionally, measuring maximum and minimum zinc concentrations under different conditions would improve the interpretation of fluorescence intensity changes.

We now include negative control in non-capacitated sperm. The data is incorporated into Figure 1B.

Line 309: "We further discovered that intracellular zinc regulates alkalinization-induced hyperpolarization in mice spermatozoa, mediated by Slo3 channel."

Additional controls are needed to substantiate this claim.

At this stage of the study, we do not have access to Slo3 knockout (KO) mice; therefore, performing additional experiments is not feasible.

Line 316: "Using FluoZin3-AM for zinc imaging, we confirmed the presence of intracellular zinc in sperm (Fig. 1A), which is consistent with previous findings (Henkel et al., 1999). Our observations revealed that treatment with capacitation medium induced a decrease in zinc fluorescence intensity (Fig. 1B, C), suggesting that zinc levels are dynamic during capacitation."

This statement must be supported by negative controls, including non-capacitated sperm conditions.

We now include negative control in non-capacitated sperm. The data is incorporated into Figure 1B.

Line 327: "We also observed that zinc chelator significantly affected the sperm motility only after, but not before, capacitation (Fig. 1-figure supplement 1)."

Data presentation should be revised to highlight the effects of capacitation itself.

The discussion should specify which motility parameters were affected and why others were not.

In the text we mentioned that:

“We incubated the isolated spermatozoa with cell permeable Zn^2+^ chelator N,N,N',N'-Tetrakis(2-pyridylmethyl)ethylenediamine (TPEN) and measured the motility parameters before and after capacitation. We found that VAP (average path velocity), VCL (curvilinear velocity), and VSL (straight-line velocity) were influenced by the TPEN treatment only after the capacitation, as shown in Fig. 1—figure supplement 1. These results demonstrate that the dynamics of zinc levels during capacitation potentially contributes to sperm motility, highlighting the importance of zinc action in sperm physiology.”

Indeed, we observed that zinc chelator significantly affected the sperm motility specifically in VAP (average path velocity), VCL (curvilinear velocity), and VSL (straight-line velocity) only after, but not before, capacitation (Fig. 1—figure supplement 1). Of note, it has been recently reported that all these motility parameters (VAP, VCL, and VSL) are reduced by Slo3-specific inhibitors in human sperm (M. Lyon et al., 2023). These findings are consistent with the idea that endogenous zinc dynamics control sperm motility through Slo3 during the capacitation process.

Figure legend is revised accordingly.

Line 369: "Structural determinants of zinc inhibition in the mSlo3 channel."

The authors should include an analysis of the evolutionary conservation of the mutated sites across Slo1, Slo2, and Slo3.

If Slo3 has a unique regulatory mechanism, these sites should show high sequence variability compared to other Slo channels.

If these sites are highly conserved, the authors should explain how Slo3 differs functionally from Slo1 and Slo2 despite this conservation.

We thank the reviewer for the valuable suggestions regarding the inclusion of additional discussion points on the structural determinants of zinc inhibition in the mSlo3 channel. We performed sequence alignment by using ClustalO between mSlo3, mSlo1, and mSlo2.2. It is worth noting that only human and frog variants of Slo2.1 sequence are available in the database, so we included only Slo2.2 subtype, as our focus was on Slo3 in mouse sperm.

Based on the alignment, E169 (mSlo3 numbering) is conserved among the Slo family channels in mice, while in contrast E205 (mSlo3 numbering) is not. To date, there have been no report examining the corresponding residues to E169 (E191 in mslo1 or E176 in mslo2.2) for their zinc sensitivity. This might be because in both channels the zinc-binding sites are well defined where they are located in RCK1 domain for Slo1 (Hou et al., 2010) and RCK2 domain for Slo2.2 (J. Zhang et al., 2023). The identified binding site in Slo2.2 is conserved in Slo2.1 but not present in Slo1 and Slo3 (J. Zhang et al., 2023), further suggesting that zinc regulation differs among Slo family members. However, this does not rule out the possibility that regions surrounding E191 or E176 could provide to additional insights into zinc regulation in these channels, which could be of interest for future studies.

Interestingly, in contrast to E169, E205 is not conserved across the Slo family, making this residue unique to the mouse Slo3 channel and potentially a determinant of zinc sensitivity in mSlo3. Given that E205 is located in the S4 domain and supported by our VCF results showing that zinc inhibition influences the motion of voltage-sensing domain of mSlo3, E205 represents an important residue to be explored in future studies. Furthermore, as this residue is unique only to Slo3, it highlights the distinct functional properties of Slo3 such as its gating mechanism as it is regulated by both membrane voltage and alkalinization, which has a different voltage range of activation compared to mSlo1 (Li et al., 2024) and involves distinct ligands and gating mechanisms compared to Slo2 (J. Zhang et al., 2023).

We add the sequence alignment results into Figure 5—figure supplement 1F.

We revised the results section as follows:

“Additionally, we performed sequence alignment by using ClustalO between mSlo3, mSlo1, and mSlo2.2. It is worth noting that only human and frog variants of Slo2.1 sequence are available in the database, so we included only Slo2.2 subtype, as our focus was on Slo3 in mouse sperm. Based on the alignment, E169 (mSlo3 numbering) is conserved among the Slo family channels in mice, while in contrast E205 (mSlo3 numbering) is not. (Figure 5—figure supplement 1F).”

We revised the discussion section as follows:

“Based on sequence alignment, E169 (mSlo3 numbering) is conserved among Slo family channels in mice, whereas E205 (mSlo3 numbering) is not (Fig. 5—figure supplement 1F). To date, no studies have examined the corresponding residues to E169 (E191 in mSlo1 or E176 in mSlo2.2) for their potential zinc sensitivity, likely because the established zinc binding sites in these channels are located in the RCK1 domain for Slo1 (Hou et al., 2010) and the RCK2 domain for Slo2.2 (J. Zhang et al., 2023). The identified zinc binding site in Slo2.2 is conserved in Slo2.1 but is absent in both Slo1 and Slo3 (J. Zhang et al., 2023), further suggesting that zinc regulation differs among Slo family members. Although regions surrounding E191 or E176 may still provide additional insights into zinc regulation and could be of interest for future investigation, E205 stands out because, unlike E169, it is not conserved across the Slo family, making it unique to mSlo3 and potentially a specific determinant of zinc sensitivity in this channel.”

Figure legend is revised accordingly.

Line 392: "Physiological relevance of zinc inhibition of the mSlo3 channel in mouse sperm."

The authors should mention the effects of zinc on CatSper channels, as CatSper is also crucial for capacitation.

Slo3 inhibition may represent only one component of zinc's broader regulatory role during capacitation.

We thank the reviewer for raising this important point regarding the physiological relevance of zinc inhibition of the mSlo3 channel in mouse sperm. We agree that we should have also discussed the effect of zinc on CatSper channels, as this channel is crucial for capacitation. To date, there are only few reports on the effect of zinc on CatSper channels, and none have been reported in mouse sperm. One important study was reported by (Jeschke et al., 2021), in which seminal zinc was found to inhibit prostaglandin-induced activation of CatSper in human sperm. The complex opposing action of seminal zinc and prostaglandins on CatSper may help preventing premature activation of CatSper in the ejaculate and act as a dilution sensor, which facilitating sperm to escape into female genital tract (Jeschke et al., 2021). Taking this into consideration, as the reviewer pointed out, zinc inhibition on Slo3 may represent only one component of zinc’s broader regulatory role during capacitation.

We added a sentence to the discussion section in the revised manuscript as follows:

“In addition to that, to date, there are only few reports on the effect of zinc on other sperm ion channels, and none have been reported in mouse sperm. One important study was reported by (Jeschke et al., 2021), in which seminal zinc was found to inhibit prostaglandin-induced activation of CatSper, a sperm-specific Ca^2+^ channel, in human sperm. The complex opposing action of seminal zinc and prostaglandins on CatSper may help preventing premature activation of CatSper in the ejaculate and act as a dilution sensor, although this study does not provide direct evidence for zinc acting directly on CatSper (Jeschke et al., 2021).”

The study presents valuable insights into the role of intracellular zinc in sperm capacitation and Slo3 channel function. However, the physiological impact of these findings remains unclear due to insufficient controls and missing key experimental data. The suggested revisions would strengthen the validity of the claims made by the authors and improve the overall scientific rigor of the manuscript.

Key Areas for Improvement:

Control experiments in non-capacitated conditions.

Increased statistical rigor in figure analyses.

More detailed experiments to confirm specificity of zinc action on Slo3.

Expanded discussion of zinc's role beyond Slo3, including CatSper regulation.

The authors should measure these effects in sperm cells using the patch-clamp technique to directly record Slo3 currents. By normalizing Slo3 currents to cell capacitance at different intracellular zinc concentrations, the authors can quantitatively assess the extent of Slo3 inhibition by zinc and strengthen the physiological relevance of their findings.

By addressing these concerns, the manuscript will provide a more robust foundation for understanding zinc's regulatory role in sperm physiology and capacitation.
